# Optimal Bladder Condition in Volumetric Modulated Arc Therapy for Prostate Cancer: The Role of Superior-Inferior Lengths of the Bladder and Dose Constraints

**DOI:** 10.7759/cureus.47148

**Published:** 2023-10-16

**Authors:** Yojiro Ishikawa, Yuki Narita, Kengo Ito, Satoshi Teramura, Takayuki Yamada

**Affiliations:** 1 Division of Radiology, Tohoku Medical and Pharmaceutical University, Sendai, JPN; 2 Department of Radiation Oncology, Southern Tohoku Proton Therapy Center, Koriyama, JPN

**Keywords:** dose-volume histogram, optimal bladder shape, prostate cancer, optimal bladder volume, volumetric-modulated arc therapy

## Abstract

Background

Optimal bladder conditions based on dose constraints in prostate cancer radiation therapy (RT) are important. In this study, the superior-inferior (SI) lengths of the bladder were assessed to define the ideal bladder state for RT.

Materials and methods

In this study, 50 prostate cancer cases treated with three-dimensional conformal radiation therapy between January and December 2021 were retrospectively analyzed. Using their CT data, a volumetric modulated arc therapy (VMAT) plan was simulated. Bladder dose constraints and dimensions, including SI, right-left (RL), and anterior-posterior (AP) lengths, were assessed. In total, 28 cases met the dose constraints and 22 cases did not meet the dose constraints.

Results

Median bladder volumes (BVs) for compliant and non-compliant cases were 163.6 ml and 88.5 ml, respectively (p<0.0001). For compliant plans, median bladder dimensions were RL: 78 mm, AP: 89 mm, and SI: 51 mm. Non-compliant plans showed RL: 72 mm, AP: 84 mm, and SI: 42 mm, with significant differences (SI: p=0.0004, RL: p=0.0065, AP: p=0.037). Established thresholds were SI: 46 mm, RL: 92 mm, AP: 75 mm, and BV: 142.8 ml. SI showed the strongest correlation with BV (coefficient: 0.78).

Conclusions

This study analyzed the SI lengths of the bladder concerning dose constraints in VMAT for prostate cancer. It was concluded that smooth treatment planning could be achieved with proper consideration of the bladder's SI distance. Further case collection and prospective studies are warranted.

## Introduction

In radiation therapy planning (RTP) for prostate cancer, it is important to set dose constraints for the nearby organs including the rectum, bladder, and femur head. If the reproducibility of the locations of these organs is affected during radiation therapy (RT), the reliability of the dose-volume histogram (DVH) at the time of RTP cannot be guaranteed [1-4]. While bone can be reproduced with fixation devices and the rectum can be addressed with defecation and drainage [5-7], there is no consensus on the optimal bladder condition other than the importance of maintaining bladder volume (BV) is crucial.

Previous studies on the optimal bladder condition for RT in prostate cancer have primarily focused on bladder volume (BV), and many studies have suggested that maintaining a BV of 100-200 ml during RT is appropriate [8-10]. There are numerous protocols for urinary storage and drinking water pretreatment [11,12], and bladder volume (BV) adjustment varies among centers [13,14]. The protocols of various clinical trials also are vague in their description of pretreatment for urination in patients with prostate cancer [15,16]. If BV is low or unstable before radiation therapy planning (RTP), repeating the CT for RTP or starting the RT with relaxed dose constraints may be necessary [9]. However, these additional procedures and tests can lead to inefficiencies in routine practice.

In routine clinical practice, some patients may have sufficient BV but still have difficulty meeting dose constraints. Prostate cancer patients may experience bladder shape changes due to prostate enlargement or overactive bladder [17,18], and these bladder shape changes may also impact the optimal bladder condition for RT in prostate cancer [19]. Although evaluation of the bladder shape before RTP could facilitate a smoother treatment process, surprisingly, there is no assessment of whether the bladder is in optimal condition before RTP.

The bladder is anatomical with a high degree of freedom in the superior-inferior (SI) direction of the bladder [19,20] Recently, several methods for predicting volume using the SI diameter of the bladder have been reported [21]. In addition, in RT for prostate cancer, the greater the SI length of the bladder is, the lower the radiation dose to the small intestine [22,23]. Therefore, we hypothesized that the optimal bladder condition of RTP for prostate cancer would be achieved by quantitatively measuring and evaluating the SI diameter of the bladder. To the best of our knowledge, there has been no study in which the relationship between quantitative evaluation of SI lengths of the bladder and dose constraint was investigated.

The aim of this study was to find an endpoint that would allow easy monitoring of optimal bladder shape and to calculate data that can be used as a basis for developing urinary protocols for future phase II and phase III trials. The primary endpoint of this study was to calculate cutoff values for the optimal bladder condition by measuring the superior-inferior lengths of the bladder. The secondary endpoint was the evaluation of the optimal BV by measured values to compare with previous reports on optimal conditions for the bladder by volume.

## Materials and methods

This retrospective study was carried out in compliance with the Declaration of Helsinki, and it was approved by the Institutional Review Board of Tohoku Medical and Pharmaceutical University. The approval number assigned to this study was 2023-2-001. We treated 50 cases with three-dimensional radiation therapy (3DCRT) for prostate cancer at our institution between January 2021 and December 2021. We investigated the electronic medical records for patients with prostate cancer in our institution and included 50 patients after RT for prostate cancer in the study. We simulated the volumetric modulated arc therapy (VMAT) plan using computed tomography (CT) data from patients treated with 3DCRT. Risk classification for prostate cancer followed the National Comprehensive Cancer Network (NCCN) risk classification.

Radiation planning

CT and magnetic resonance imaging (MRI) were performed for all patients, and MRI and CT images were superimposed for treatment planning. The radiation oncologist registered the regions of interest of the clinical target volume (CTV), planning target volume (PTV), and risk organs (rectum, bladder, and femoral head) during treatment planning. For low-risk cases according to the NCCN risk group, CTV included the prostate alone. CTV included the base of the seminal vesicle for the intermediate-risk group, and for T3b cases, the whole seminal vesicle was included as CTV. The CTV was expanded in three dimensions with a 0.8-cm margin to obtain the planning target volume (PTV) except for the prostate-rectum interface, where a 0.5-cm margin was adopted to decrease rectal involvement. The rectum, bladder, bowel, and femur were contoured as critical normal tissue structures. The contouring details followed the report by Ishikawa et al. [24], but the rectum and bladder were contoured without setting the wall area, and the region was created as a solid area. Patients were treated in the supine position. Neither urinary catheters, rectal balloons, gold fiducial markers, nor hydrogel spacers were utilized. Additionally, before acquiring the treatment planning CT images, each patient was asked to urinate 30 minutes prior. No specific instructions were provided about water intake; it was left to the patient's discretion.

Volumetric modulated arc therapy

Fifty prostate cancer patients who were treated in our hospital were studied. All of the plans contained one or two full arcs. Each plan was generated in Raystation treatment planning system (RaySearch Laboratories AB, Stockholm, Sweden). All prostate plans were planned for a 10 MV X-ray beam on a Synergy linear accelerator (Elekta Ltd., Crawley, UK). When planning by VMAT for analysis, treatment plans were made by medical physicists, and the treatment plans had to meet the treatment plan evaluation criteria of our institution (Table 1). The prescribed dose used to cover 95% of the target volume (D95) was 74 Gy [2,25]. The maximum dose heterogeneity allowable in the planning target volume (PTV) was 10%. In the present study, we compared the V50, V60, V65, and V70 evaluations for each bladder volume after the treatment plan was finalized and also evaluated the achievement rate of the treatment plan evaluation criteria for the bladder.

**Table 1:** Dose constraints of the volumetric modulated arc therapy plan D95; Dose received by 95% of volume, V75 (the volume of the rectum receiving at least 75% of the prescribed radiation dose), V70 (at least 70% dose), V60 (at least 60% dose), V50 (at least 50% dose) and V40 (at least 40% dose). V70 (the volume of the bladder receiving at least 75% of the prescribed radiation dose), V65 (at least 65% dose), V60 (at least 60% dose), and V50 (at least 50% dose).

		Dose constraints
PTV	D95	74 Gy
	Mean	<77.7 Gy (<105%)
	Maximum	<81.4 Gy (<110%)
Rectum	V75	<5%
	V70	<20%
	V60	<40%
	V50	<50%
	V40	<65%
	Maximum	<78 Gy
Bladder	V70	<20%
	V65	<25%
	V60	<35%
	V50	<50%
Femoral heads	Maximum	<50 Gy
Small intestine	Maximum	<60 Gy
Sigmoid colon	Maximum	<65 Gy
Body	Maximum	<81.4 Gy

Statistics

**Figure 1:**
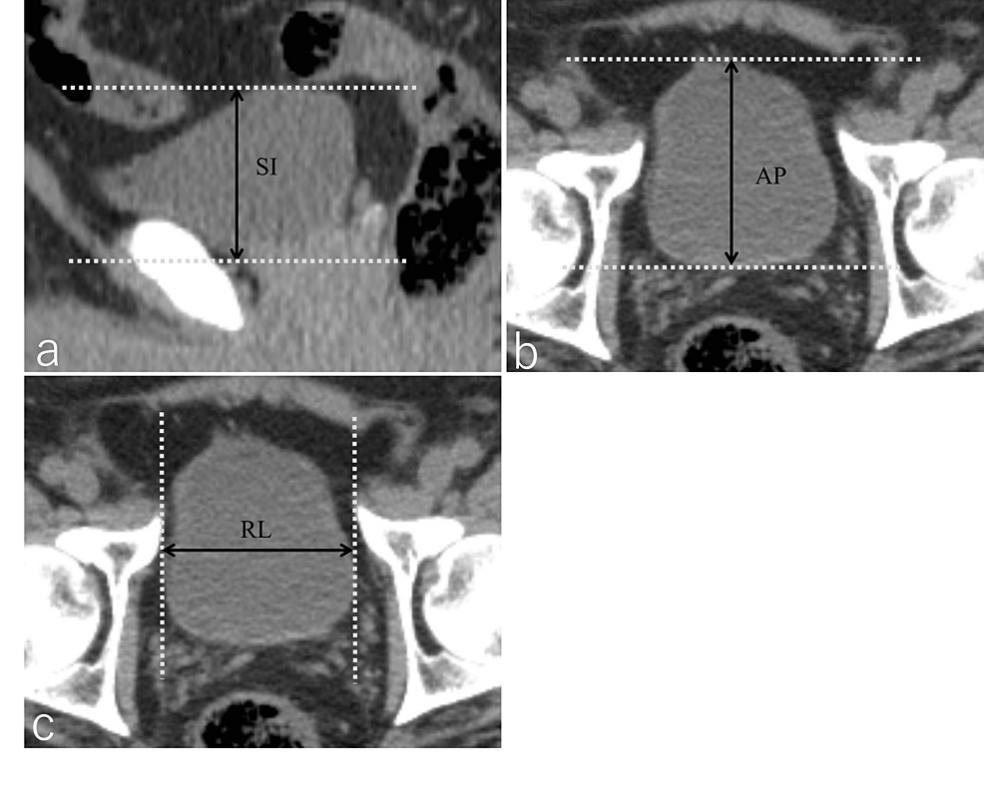
Image of bladder measurement using CT for radiation therapy planning We measured the bladder lengths with the largest width of SI, AP, and RL where SI =superior-inferior length of the bladder (a), AP = anterior-posterior length of the bladder (b), and RL =right-left length of the bladder (c). SI of the bladder was determined by using sagittal images. Axial images were used for AP and RL.

Statistical analyses were performed with JMP v.16 (SAS Institute Inc., Cary, NC). Continuous variables were presented as medians (range). We measured the bladder lengths with the largest width of RL, AP, and SI, where RL=right-left length of the bladder, AP=anterior-posterior length of the bladder, and SI=superior-inferior length of the bladder (Figure 1).

The correlations of BV with RL, AP, and SI were analyzed using Pearson's correlation coefficients. In order to calculate the cutoff values for bladder lengths of RL, AP, SI, and BV, receiver operating characteristics (ROC) analysis was used to determine the sensitivity and specificity of the test results. Maximum sensitivity and maximum specificity were used to calculate the cutoff values. The chi-square test was used to compare various clinical factors including age, NCCN risk group, stage, and prostate-specific antigen (PSA) value. Logistic regression analysis was performed to examine the correlations between these clinical factors. A p-value of <0.05 was deemed statistically significant.

## Results

The BV of all cases ranged from 47.4 to 356.6 ml with a median of 132.6 ml. Of the examined cases, 28 treatment plans adhered to the bladder dose constraints: V50≤50%, V60≤35%, V65≤25%, and V70≤20%. Twenty-two cases surpassed the constraints: V50>50%, V60>35%, V65>25%, and V70>20%. The median bladder volumes were 163.6 ml for the compliant cases and 88.5 ml for the non-compliant cases, and the difference was significant (p<0.0001).

In all cases, the median bladder dimensions were 76 mm for RL (range: 61-97 mm), 86 mm for AP (range: 61-123 mm), and 47 mm for SI (range: 26-70 mm). In the 28 plans adhering to the constraints, the median bladder dimensions were 78 mm for RL (range: 65-97 mm), 89 mm for AP (range: 74-123 mm), and 51 mm for SI (range: 28-70 mm). In the 22 plans that surpassed constraints, the median bladder dimensions were 72 mm for RL (range: 61-91mm), 84 mm for AP (range: 61-91mm), and 42 mm for SI (range: 26-54mm). The differences were statistically significant with p=0.0065 for RL, p=0.037 for AP, and p=0.025 for SI. Correlations with clinical factors are itemized in Table 2.

**Table 2:** Patient characteristics and bladder measurements AP, anterior-posterior length of bladder; HT, hormone therapy; RL, right-left length of bladder; NCCN, National Comprehensive Cancer Network; PSA, prostate-specific antigen SI, superior-inferior length of bladder

	Within-Constraint Group	Exceeding-Constraint Group	p-value
No. of patients	28	22	
Age (years)	58-79	55-78	0.72
Median (range)	72 (55-78)	72 (59-78)	
NCCN risk			
Low	2 (7.1%)	3 (13.4%)	0.53
Intermediate	8 (28.5%)	5 (22.7%)	
High	18 (64.3%)	14 (63.6%)	
PSA (mg/ml)			
Median (range)	7.2 (3.97-417)	7.83 (2.17-375.8)	0.52
T stage			
T1	7 (25.0%)	3 (13.6%)	0.62
T2	12 (42.9%)	14 (63.6%)	
T3a	6 (21.4%)	3 (13.6%)	
T3b	3 (10.7%)	1 (4.6%)	
T4	0	1 (4.6%)	
Gleason score			
6	6 (10.7%)	2 (9.1%)	0.62
7	11 (39.2%)	10(54.4%)	
8	9 (32.1%)	6 (27.3%)	
9	5 (17.9%)	4 (18.2)	
10	0	0	
HT			
with	26 (92.8%)	19(86.3%)	0.28
without	2 (7.1)	3 (13.6%)	
Median (range)	78 (65-97)	72 (61-92)	0.0065
AP (mm)			
Median (range)	89 (74-123)	84 (61-91)	0.037
SI (mm)			
Median (range)	51 (28-70)	42 (26-54)	0.025
Bladder volume (ml)			
Median (range)	163.6 (68.7-356.5)	88.5 (47.4-152.0)	<0.0001
Prostate volume (ml)			
Median (range)	24.8 (14.5- 126.2)	31.3 (15.5-60.4)	0.14
Planning target volume (ml)			
Median (range)	107.4 (66.0-152.1)	112.1 (45.0-170.1)	0.37

The threshold for SI was established at 46 mm, an area under the ROC curve (AUC) value of 0.75 (p=0.001). The thresholds for RL and AP were 92 mm and 75 mm, respectively with AUC values of 0.74 and 0.72, respectively. The BV threshold was pinpointed at 142.8 ml, yielding an AUC value of 0.88. Detailed results regarding the thresholds are shown in Table 3.

**Table 3:** The cut-off values to meet the dose constraints for bladder calculated based on the receiver operating characteristic curve AP, anterior-posterior length of bladder; RL, right-left length of bladder; SI, superior-inferior length of bladder

Parameter	cutoff	AUC	p-value
RL (mm)	92	0.74	0.0003
AP (mm)	75	0.72	0.0097
SI (mm)	46	0.75	0.001
Bladder volume (ml)	142.8	0.88	0.0001

**Figure 2:**
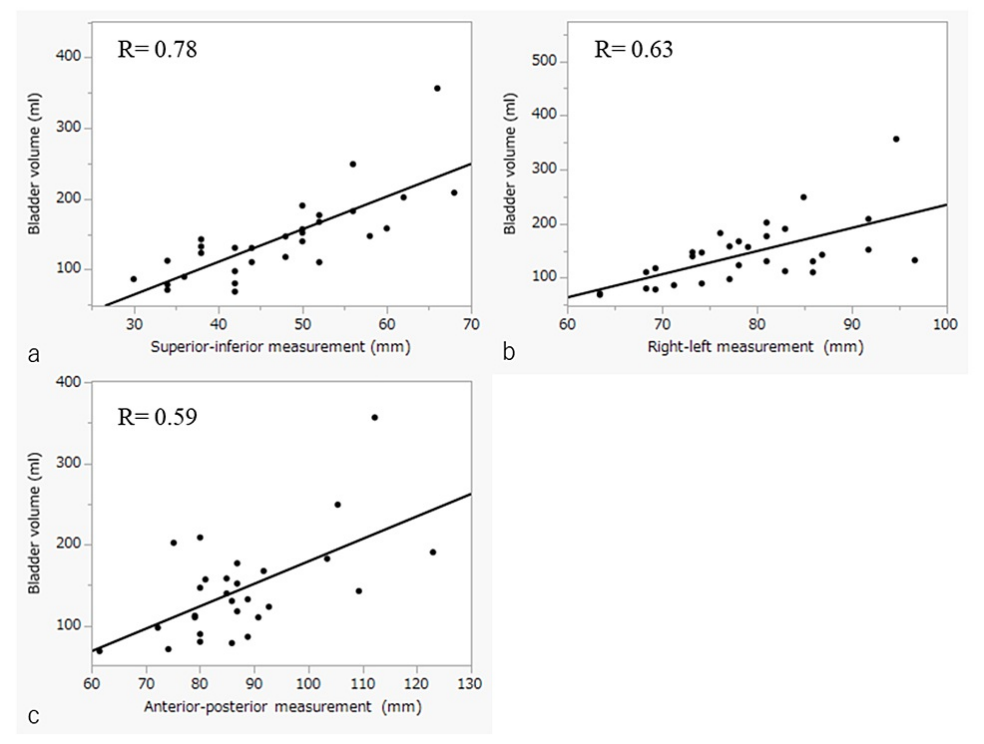
Correlations between bladder volume and measurements Correlations between bladder volume and measurements: correlation between superior-inferior length of the bladder and bladder volume (a), correlation between right-left length of the bladder and bladder volume (b) and correlation between anterior-posterior length of the bladder (c).

Bladder dimensions in SI, RL, and AP directions were correlated with coefficients of 0.78, 0.63, and 0.59 when juxtaposed with the actual BV (Figure 2).

Both univariate and multivariate evaluations of clinical factors and bladder measurements, segregated by compliant cases and non-compliant cases, were executed. The univariate assessment showed significant variances in RL and SI bladder dimensions (p=0.0022 and p=0.0004, respectively). Multivariate scrutiny highlighted the pronounced significance of SI's length (OR: 36.94; interval: 2.85-478.51, p<0.0004). Other parameters including age, risk bracket, T classification, PSA metrics, and Gleason score did not register any consequential distinctions. For a comprehensive view, refer to Table 4.

**Table 4:** Univariate and multivariate analysis AP, anterior-posterior length of bladder; HT, hormone therapy; RL, right-left length of bladder; NCCN, National Comprehensive Cancer Network; PSA, prostate-specific antigen SI, superior-inferior length of bladder

	Univariate			Multivariate		
Parameter	OR	95% CI	p-value	OR	95% CI	p-value
Age						
Continuous variable	0.93	0.82-1.05	0.26			
NCCN						
High risk	1					
Low-intermediate risk	0.57	0.13-2.50	0.45			
T stage						
T3-T4	1					
T1-T2	0.73	0.15-3.49	0.69			
Gleason score						
>7	1					
≤7	0.57	0.13-2.50	0.45			
PSA						
Continuous variable	1.04	0.98-1.08	0.23			
HT						
with	1					
without	0.29	0.026-3.12	0.30			
AP						
Continuous variable	1.10	1.00-1.21	0.013	1.09	0.92-1.28	0.16
≥92 mm	1					
<92 mm	7.8	0.80-75.60	0.081			
RL						
Continuous variable	1.16	1.03-1.31	0.0041	1.17	0.91-1.51	0.20
≥75 mm	1					
<75 mm	9.3	1.51-57.6	0.0022	8.5	0.70-104.07	0.092
SI						
Continuous variable	1.28	1.08-1.51	<0.001	1.09	0.92-1.29	<0.0001
≥46 mm	1					
<46 mm	39	3.8-40.0	0.0004	36.94	2.85-478.51	0.0004

## Discussion

In this study, we investigated whether the SI length of the bladder can define the optimal bladder condition to meet dose constraints for the bladder. We confirmed that it is desirable to plan with an SI bladder length of at least 46 mm to meet the dose constraints of the simulated plan by VMAT for prostate cancer. The reason why the length in the SI direction is an indicator is presumably that the bladder is attached ventrally and surrounded by soft tissue, but the SI direction, especially the head side, allows greater freedom of bladder extension [19,20]. There has been no study in which a reference value was calculated. Notably, the results of this study indicated that the correlation between BV and the SI direction is stronger than the correlations of BV with the RL and AP directions.

Ultrasound imaging or cone beam CT (CBCT) is a minimally invasive procedure and for evaluating BV during RT. However, for ultrasound imaging, there is variation in test results based on the operator’s technique [26]. CBCT cannot be utilized prior to treatment planning, and there is currently no implemented application for measuring bladder volume just before the treatment session. It is reasonable to ascertain BV at the time of treatment planning using CT imaging by performing a simple bladder measurement such as measurement of the SI diameter of the bladder.

Before the present study, BV was evaluated on RTP to define the optimal BV in several institutions. Nakamura et al. found 150 ml to be a reasonable pretreatment bladder capacity [9]. The most commonly reported values are 100-200 ml [8-10]. However, in those studies, the residual urine volume is divided into groups by every 50 ml. There is no mention in the reports of why it was necessary to classify groups by 50 ml. The cutoff value is slightly higher than that in the current study because the evaluation is based on 50-ml units. Although the main purpose of this study was not to calculate the optimal BV, we attempted to add statistical analysis to those previous reports for BV and to establish a cutoff value using ROC curves. The cutoff value of BV was 142.8 ml and this AUC value was as high as 0.88. We believe that the ROC curve provides a clearer cutoff value for BV. It was confirmed that optimal treatment planning might be possible even with BV less than 150 ml.

BV during RT can exhibit significant fluctuations. One study conducted in 419 patients showed that BV determined by CT imaging varied between 41.0 and 1501.3 cm³ [27]. Another study showed that BV measurements on CT scans prior to RT were within the range of 70-509 cm³ with an average value of 187 cm³ [28]. In another study, despite being advised to keep their bladders full, 54% of a group of 24 patients experienced a pretreatment bladder volume decrease of at least 50% with a mean reduction of 124 cm³ [29]. In our analysis, BV ranged between 47.4 and 356.6 ml with a median value of 132.6 ml. The BV recorded in this study was generally smaller than the values in earlier works. This difference might be due to our protocol that did not require patients to drink water before treatment and due to the treatment planning CT being performed only 30 minutes after urination. The difference might due to the older age of our cohort, with a median age of 72 years. Prostate cancer is more common in the elderly, and many patients with prostate cancer also suffer from benign prostatic hyperplasia [25]. The cutoff value of 142.8 ml for BV in this study might be a useful cutoff value when determining BV in elderly patients with prostate cancer.

**Figure 3:**
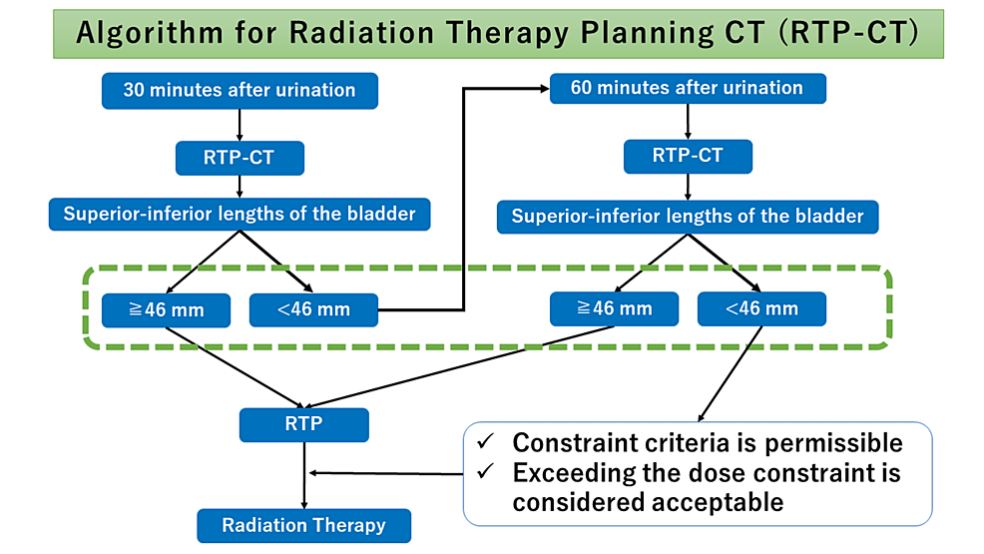
An algorithm showing the process of radiation therapy planning CT (RTP-CT). The bladder's superior-inferior length is initially measured 30 minutes after urination. If the length is less than 46 mm, RTP-CT is performed again 60 minutes after the initial urination. If measurements consistently remain below 46 mm, radiation therapy planning is adjusted with a relaxation of dose constraints.

Based on the results of this study, if the SI lengths less than 46 mm, patients should be advised to retain urine to achieve an SI length greater than 46 mm. Only once a diameter of 46 mm or more is confirmed would they proceed with the treatment plan. Figure 3 shows how our hospital practically employs these research-based measurements in clinical setting.

The present study has some limitations. First, this was a retrospective study and the number of cases (50) was small. However, in most previous studies, there were only about 20 to 30 cases in each group [9,10]. Therefore, the number of patients in this study is not small compared to the numbers in previous studies. Another limitation of this study is that clinical findings related to irradiation do not include information on urinary status. We did not use a protocol for drinking water in this study. Although there are many institutions that have established protocols for drinking water, the relationship between the amount of water consumed and bladder volume is unclear, and it is stated in many reports that there is no effect of drinking water until 1-2 hours after consumption [30]. Finally, for VMAT with conventional fractionated irradiation, D50 and Dmean prescriptions are common and recommended by the International Commission on Radiation Units and Measurements (ICRU), but we used the D95 prescription in this study. 

## Conclusions

This study analyzed the SI lengths of the bladder concerning dose constraints in VMAT for prostate cancer. It was concluded that smooth treatment planning could be achieved with proper consideration of the bladder's SI distance. Further case collection and prospective studies are warranted.
